# Long Non-Coding RNA *FENDRR*: Gene Structure, Expression, and Biological Relevance

**DOI:** 10.3390/genes12020177

**Published:** 2021-01-27

**Authors:** Przemyslaw Szafranski, Paweł Stankiewicz

**Affiliations:** Department of Molecular and Human Genetics, Baylor College of Medicine, Houston, TX 77030, USA; pawels@bcm.edu

**Keywords:** regulatory RNA, ceRNA, divergent genes, lncRNA enhancer, lung development, fibrosis, drug resistance

## Abstract

The *FOXF1* Adjacent Noncoding Developmental Regulatory RNA (*Fendrr*) plays an important role in the control of gene expression in mammals. It is transcribed in the opposite direction to the neighboring *Foxf1* gene with which it shares a region containing promoters. In humans, *FENDRR* is located on chromosome 16q24.1, and is positively regulated both by the *FOXF1* distant lung-specific *cis*-acting enhancer and by *trans*-acting FOXF1. *Fendrr* has been shown to function as a competing endogenous RNA, sponging microRNAs and protein factors that control stability of mRNAs, and as an epigenetic modifier of chromatin structure around gene promoters and other regulatory sites, targeting them with histone methyltrasferase complexes. In mice, *Fendrr* is essential for development of the heart, lungs, and gastrointestinal system; its homozygous loss causes embryonic or perinatal lethality. Importantly, deregulation of *FENDRR* expression has been causatively linked also to tumorigenesis, resistance to chemotherapy, fibrosis, and inflammatory diseases. Here, we review the current knowledge on the *FENDRR* structure, expression, and involvement in development and tissue maintenance.

## 1. Introduction

Nearly the entire human genome is transcribed; however, only approximately 2% of the transcriptome becomes translated into polypeptides longer than 100 amino acids [1,2]. Long non-coding RNAs (lncRNAs) are classified as transcripts of more than 200 nt in length, showing a very limited translational potential. Like mRNAs, most lncRNAs are synthesized by RNA polymerase II and can be capped, polyadenylated, or spliced. LncRNAs have been found in the nucleus and/or cytoplasm. They can functionally interact with microRNAs (miRs), mRNAs, dsDNA, or proteins [3,4].

A subset of anti-sense lncRNAs have their 5′ ends located in proximity to the 5′ ends of the protein-coding genes transcribed in the opposite direction. In some cases, those divergent genes may overlap (e.g., *RNF157-AS1* and *FOXJ1*, *FOXC2-AS1* and *FOXC2*, *ZCCHC14-DT* and *ZCCHC14*). In other cases, like lncRNA gene *FENDRR* (*FOXF1* Adjacent Noncoding Developmental Regulatory RNA; HGNC: 43894, MIM: 614975) and the transcription factor (TF)-coding *FOXF1*, they share a genomic region containing promoters. The symbol *FENDRR* is an acronym for the Fetal-lethal Non-coding Developmental Regulatory RNA and reflects the significance of this lncRNA in early embryonic development [5]. Previously, *FENDRR* was referred to as *FOXF1-AS1*, *lincFOXF1*, *onco-lncRNA-21*, or *TCONS_00024240. FENDRR* was identified in the studies that expanded the catalog of human lncRNAs to over 3000 based on their (i) distinctive histone 3 (H3)-based chromatin signature (H3K4me3-H3K36me3) that marks transcribed genes and (ii) limited protein coding potential [6].

Here, we review the current knowledge on this important but still relatively poorly understood regulatory lncRNA, including the most recent studies on the control of its expression and potential targets in the developing lungs.

## 2. *FENDRR* Structure

**Figure 1:**
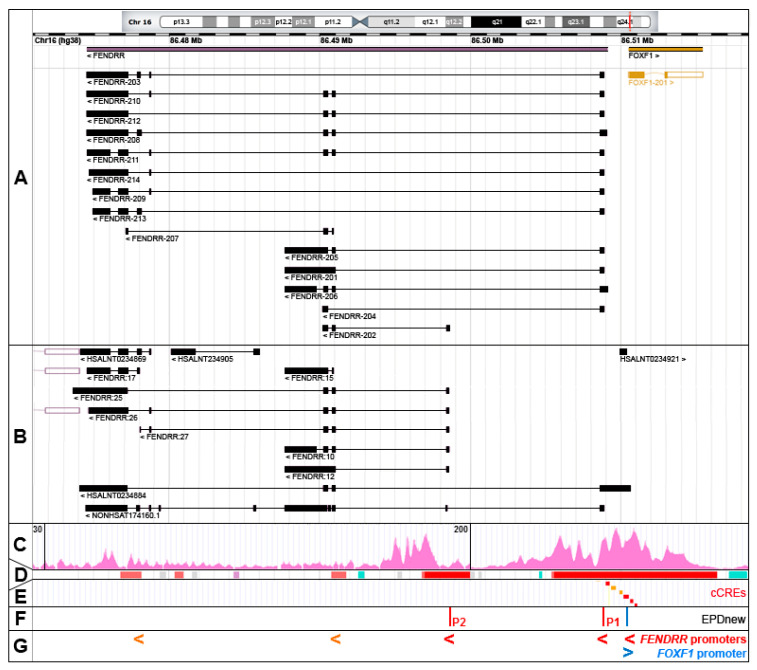
The *FOXF1* Adjacent Noncoding Developmental Regulatory RNA (*FENDRR*) gene, alternatively spliced *FENDRR* transcripts, and promoters. (**A**) GENCODE annotated *FENDRR* isoforms (RNAcentral browser screenshot). (**B**) Selected additional *FENDRR* isoforms listed in RNAcentral. (**C**) Chromatin histone 3 modification, H3K4me3, usually found around active promoters (ENCODE ChIP-seq data from human lung fibroblasts, HLFs). (**D**) Ensembl-annotated promoters (red) and promoter flanking regions (orange). (**E**) ENCODE candidate *cis*-regulatory elements (cCREs, promoters are in red). (**F**) Eukaryotic promoter database (EPDnew)-annotated promoters. (**G**) Compilation of *FENDRR* promoters: EPDnew-annotated, cCRE promoters (red) and cCRE promoter flanking regions (orange). Note that promoter locations correlate with 5′ ends of the majority of *FENDRR* isoforms.

In humans, *FENDRR* maps to chromosome 16q24.1 at chr16:86,474,529-86,509,099 (GRCh38; ENSG00000268388, RNAcentral: http://rnacentral.org, [7]) (Figure 1A). However, due to a variety of *FENDRR* transcription start sites (TSS) and alternative splicing, the combined genomic region coding for all *FENDRR* transcripts is actually larger, ~37 kb in size, mapping between chr16:86,473,637 for the 3′ end of the *FENDRR:25* transcript and chr16:86,510,615 for the 5′ end of the *HSALNT0234884* transcript (LncBook: http://bigd.big.ac.cn/lncbook, [8]) (Figure 1A,B). Due to the presence of two to six (10 in the *NONHSAT174160.1* isoform) relatively short exons, *FENDRR* isoforms are only ~0.4–4 kb in size after splicing. LncBook, a curated database of human lncRNAs, incorporated in a non-coding RNA database RNAcentral, lists 50 *FENDRR* transcripts (examples are shown in Figure 1A,B). Fourteen of them are annotated in the GENCODE database (http://www.gencodegenes.org, [9]) as *FENDRR-201* to *214* (Figure 1A). The diversity of *FENDRR* splicing contrasts with that of the neighboring protein-coding *FOXF1* gene that features only a single isoform in humans, and supports a contention [10] that non-coding genes undergo alternative splicing more often than the protein-coding genes.

*FENDRR* has been predicted to have a very low coding capability based on PRIDE reprocessing 2.0 (=0), PhyloCSF score (=65.5245), CPAT coding probability (=28.39%), and Ribosome-profiling: Lee translation initiation sites (=0) and Bazzini small ORFs (=0) predictions (LNCipedia: http://lncipedia.org). In contrast to evolutionary conservation of *FOXF1*, the orthologs of human *FENDRR* are best conserved among higher primates and have not been identified in animals other than mammals (http://genome.ucsc.edu/ENCODE).

## 3. *FENDRR* Transcription

### 3.1. Promoter

The chromatin hallmarks of a human promoter include (i) a nucleosome-free region around and upstream of a transcription start site (TSS), (ii) a peak of RNA polymerase II binding slightly downstream of TSS and usually overlapping with the TF binding sites, and (iii) several H3K4me3-marked nucleosomes especially in the downstream portion of the promoter region [11]. Taking into account these criteria, *FENDRR* transcription can start from at least three promoters. The majority of the *FENDRR* isoforms are transcribed from the intergenic promoter P1, annotated in the Eukaryotic promoter database (EPDnew, http://epd.vital-it.ch, [11]) as a promoter element mapping at chr16:86,508,876–86,508,935 and in ENCODE as candidate *cis*-regulatory element (cCRE) mapping at chr16:86,508,968–86,509,250 (Figure 1C–F). This promoter is located within a large island of 365 CpGs and is sensitive to DNA methylation. It belongs to a class of promoters with a dispersed TSS pattern. The emerging view on the functioning of human promoters is that they are intrinsically bi-directional and their actual directionality is controlled both at the transcriptional and/or post-transcriptional levels [12,13,14,15,16]. In support of this notion, an abortive transcription (the *HSALNT0234921* transcript mapping to chr16:86,510,010–86,510,341) originates possibly from this promoter in the direction opposite to the *FENDRR* transcription (Figure 1B). The *FENDRR* promoter with the apparently second highest usage, P2 (EPDnew-annotated element: chr16:86,498,542–86,498,601), is located within *FENDRR* intron 1 and may initiate *FENDRR-202*, *FENDRR:10* to *12*, *26*, *27*, and *29* transcripts (Figure 1). As in the case of the intergenic promoter, the intragenic *FENDRR* promoter P2 is located within an island of 184 CpGs. It is possible that *FENDRR-207*, and *FENDRR:14* to *17* transcripts initiate from two other intragenic promoters located further downstream (Figure 1). Interestingly, one of the *FENDRR* isoforms, the *HSALNT0234884* transcript (chr16:86,474,121–86,510,614), begins with a cCRE (chr16:86,510,620–86,510,824) overlapping the 5′ end of the non-coding portion of the *FOXF1* exon 1 (Figure 1).

### 3.2. Enhancer

**Figure 2:**
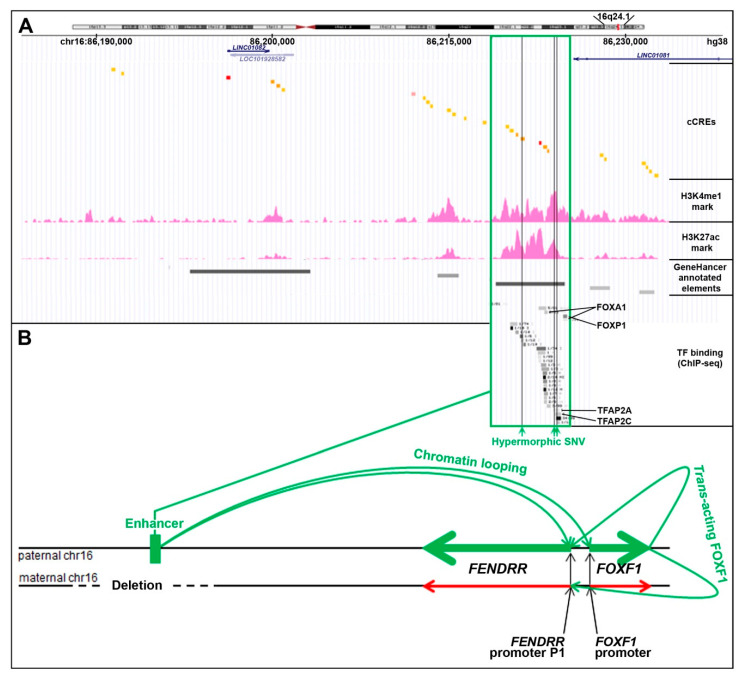
*Cis*- and *trans*-regulation of the *FENDRR* expression in the lungs (modified from [24]). (**A**) *FENDRR-FOXF1* distant enhancer region, located ~250 kb centromerically to the 3′ end of *FENDRR* (UCSC genome browser screenshot). The most essential part of this enhancer is shown in green frame. The enhancer features histone 3 modifications, usually found in active enhancers, and ChIP-seq-determined in HLFs binding sites for numerous transcription factors (TF): FOXA1, FOXP1, CEBPB, MAFK, RAD21, SMARCC1, CTCF, GTF2F1, KAP1, TBP, JUNs, EP300, STAT3, FOS, TFAP2A, and TFAP2C (ENCODE). Single nucleotide variants (SNVs, vertical green lines) that map to the essential part of the enhancer have been proposed to increase activity of the undeleted allele of the enhancer and mitigate ACDMPV phenotype in patients with heterozygous CNV deletions of the enhancer. (**B**) Scheme of mono-allelic expression of *FENDRR* from the paternally inherited chromosome 16 in the presence of a heterozygous CNV deletion of the maternal allele of the *FENDRR-FOXF1* enhancer. The drawing shown is not to scale.

The lung-specific *FOXF1* enhancer is located ~270 kb upstream to the *FOXF1* gene [17,18,19,20,21]. This enhancer was originally described as ~60 kb-large regulatory region based on the overlap of heterozygous copy number variant (CNV) deletions detected in patients with Alveolar Capillary Dysplasia with Misalignment of Pulmonary Veins (ACDMPV, MIM: 265380) due to *FOXF1* haploinsufficiency [17]. It consists of six regulatory elements annotated as enhancers in GeneHancer database [22], each overlapping several cCREs (Figure 2A). In cultured human lung fibroblasts (HLFs) these regulatory elements feature H3K27ac and H3K4me1 chromatin modifications which are the predominant H3 marks at the nucleosomes flanking the active/poised enhancer elements (ENCODE). Moreover, chromosome circular conformation capture (4C) analysis in human pulmonary microvascular endothelial cells [17] and 3C analysis in cells isolated from mouse embryonic lungs [19] showed that this distant enhancer physically interacts with the *FENDRR-FOXF1* intergenic promoter region. Interestingly, we have found a ~35 kb-large genomic instability hotspot, featuring the evolutionarily young LINE1 elements, L1PA2 and L1PA3 flanking five *Alu* repeats, located at the distal edge of this enhancer region, and responsible for several pathogenic enhancer deletions of which the distal breakpoints are mapped within the hotspot [23]. The appearance of this genomic instability hotspot in the course of evolution correlates with the branching out of the *Homo-Pan-Gorilla* clade.

Based on the overlap of additional pathogenic CNV deletions causative for ACDMPV and the presence of hypermorphic single nucleotide variants (SNVs) in the undeleted allele of the enhancer that significantly ameliorated the lethal ACDMPV phenotype by increasing *FOXF1* expression, this enhancer was narrowed to the ~10 kb-large most essential region [20,21]. This narrowed interval harbors a GeneHancer-annotated regulatory element GH16J086219 (chr16:86,218,986–86,224,837, overlapping with seven cCREs) that corresponds to one of the super-enhancers proposed by Hnisz et al. [25] based on their analysis of the H3K27ac ChIP-seq data from a spectrum of human cell types including fetal lung fibroblasts IMR-90.

Recently, through a correlation of the parental origins of chromosome 16, bearing the heterozygous CNV deletions of this enhancer with that of the transcribed *FENDRR* allele, we have found that the *FOXF1* enhancer regulates *in cis* also *FENDRR* [24] (Figure 2B). This finding may help explain the results of in-depth expression analyses of mouse *Fendrr/Foxf1* and other lncRNA/protein-coding divergent gene pairs showing that lncRNAs mimic the expression patterns of their protein-coding neighbors [26,27].

Besides the *FENDRR/FOXF1* distant enhancer, there are several other regions upstream or downstream to TSS of *FENDRR* (within a large GeneHancer-annotated element GH16J086491) that, based on their H3 chromatin signature and eQTLs (expression quantitative trait loci), may potentially function as *FENDRR* proximal enhancers or modulators of its tissue specificity. For instance, in mice, a genomic region located ~1 kb downstream of *Foxf1* functions as *Foxf1* enhancer in foregut mesoderm and mesenchyme of developing liver and lungs [28]. It was also shown by 3C, using mouse lung cells, that this regulatory element physically interacts *in vivo* with *Fendrr*-*Foxf1* intergenic region [19].

### 3.3. Regulation of FENDRR by FOXF1 

In contrast to the co-regulation of *FENDRR* and *FOXF1* expression by the same *cis*-acting distant enhancer, the involvement in this regulation of the *trans*-acting TF FOXF1 was unexpected. Depletion of *FOXF1* in lung fibroblasts by siRNA or *FOXF1* point mutations causative for ACDMPV were found to correlate with a substantial (~50%) decrease in *FENDRR* levels (measured by qPCR with TaqMan assay for the exon 1/2 junction, and RNA-seq, respectively) [24]. *Foxf1* was also shown to likely support *Fendrr* expression in mice [29]. However, the binding of *FOXF1* to the *FENDRR* promoters or the enhancer has yet to be documented. Interestingly, *FENDRR* promoters and the enhancer all contain several variants of the FOX TF binding RYAAAYA motif (R = purine, Y = pyrimidine; [30]), suggesting a possibility of direct regulation of *FENDRR* expression by *FOXF1*. In support of this notion, ChIP-seq experiments of the TF binding (ENCODE) showed that other members of the FOX TF family, especially FOXA1, bind to the *FENDRR* major promoter, P1, and the essential region of the enhancer. In addition, *FOXF1* might also indirectly regulate *FENDRR* expression through the control of factors that directly regulate *FENDRR*. Based on our RNA-seq analyses of the ACDMPV transcriptomes, of about 40 TFs potentially interacting, based on ENCODE’s ChIP-seq data, with the *FENDRR* primary P1 promoter and the most essential portion of the enhancer, the expression of a histone methyltransferase subunit, ASH2L (binding next to *FENDRR* P1 promoter’s cCRE), positively correlates with the expression of *FOXF1* (reduced by ~40% in ACDMPV cases linked to *FOXF1* deficiency) [24]. ASH2L can interact with MLL [31], a Trithorax-group (TrxG) protein involved in histone 3 methylation, H3K4me3, which is usually associated with open chromatin at active promoters. Thus, ASH2L might mediate positive regulation of *FENDRR* expression by *FOXF1*.

### 3.4. Other Factors Controlling FENDRR Transcription

Another potential regulator of *FENDRR* expression is a pro-apoptotic Annexin 2 (ANXA2) [32]. In electrophoretic mobility shift assay performed using extracts from rat pancreatic acinar cells and fragments of the *Fendrr* promoter, ANXA2 was specifically bound to the *Fendrr* intergenic promoter, and the increase in *Anxa2* expression in caerulein-treated pancreatic cells positively correlated with the increase in the *Fendrr* level.

Using a reporter assay in lung cancer cells lines, *FENDRR* was also shown to be positively regulated by EGR2 and TFAP2A [33]. Both these TFs are known to bind to the *FENDRR* primary promoter based on ChIP-seq data from lung fibroblasts (ENCODE).

Interestingly, induced expression of *Mesp1* during cardiomyocyte differentiation also led to upregulation of several genes including *Fendrr* [34]. Thus, *Fendrr* can be a downstream effector of MESP1, a TF best known as a master regulator of cardiovascular system development.

Regarding suppressors of *FENDRR*, its expression is negatively regulated in the lungs by SMAD3 (but not SMAD2), which is a major signal transducer for cell-membrane Ser/Tyr kinase receptors of TGF-β1 [35].

Of note, using ChIP-seq for TBX2 and TBX4 in IMR-90 fibroblasts, we have found a specific binding of (i) TBX4 at chr16:86,223,833–86,225,160, largely overlapping the GH16J086219 regulatory element in the essential region of the *FENDRR-FOXF1* enhancer, (ii) TBX4 at chr16:86,508,364–86,508,853 next to the *FENDRR* primary promoter, and (iii) TBX2 at 86,507,893–86,509,510, overlapping the *FENDRR* primary promoter [36]. The functional significance of these interactions is currently unknown, but they suggest the existence of a direct regulatory relationship between TBX4-FGF10 and SHH-FOXF1 signaling pathways during lung development.

Lastly, it has been suggested that *FENDRR* expression can be regulated through the epigenetic modification of its promoter (P1) that overlaps a large CpG island. The hypermethylation of this promoter has been shown to correlate with suppression of *FENDRR* expression in gastric cancer-associated fibroblasts [37]. Hypermethylation of the *FENDRR* promoter was also found in 36% of non-small cell lung cancers [38].

## 4. Mechanisms of *FENDRR* Functioning

### 4.1. Competing Endogenous RNA (ceRNA)

The well-known activity exhibited by lncRNAs is to function as a molecular sponge. *FENDRR* was shown to sequester, by sequence complementarity, at least ten different miRs, resulting in an increase in the expression of genes whose mRNAs were targeted for degradation by those miRs (Table 1). The majority of miR binding sites in *FENDRR* have been mapped to the middle of the gene, in the region corresponding to the exon 4 of *FENDRR-206* (RefSeq transcript variant 2) (Table 1, Figure 1A).

Importantly, ceRNA function may be controlled by alternative splicing. For instance, *FENDRR-203*, *209*, *213* and *214* do not have exons corresponding to the exons 3 and 4 of *FENDRR-206* that bind several miRs (Table 1). Likewise, *FENDRR-207* cannot bind *miR-184*, whereas *miR-126* can be bound by the 5′ extension of the exon 1 present only in *FENDRR-206* or *208*. In mice, *miR-106b* binds to the largest exon 4 of MS-*Fendrr-202*, but likely not to the isoforms MS-*Fendrr-201* or MS-*Fendrr-203* in which the portion of exon 4 bearing *miR-106b* binding site is spliced out.

Besides interacting with miRs, *FENDRR* can directly and specifically bind to 3′ untranslated regions (UTRs) of mRNAs. In particular, *FENDRR* was shown to compete with the Hu/ELAV-like protein 1 (HuR) for binding to the AU-rich elements in the 3′UTR of the multi-drug resistance gene *MDR1* (*ABCB1*) mRNA [44]. This interaction prevents binding of HuR to the *MDR1* mRNA 3′UTR, destabilizing *MDR1* mRNA. As expected, the overexpression of *FENDRR* in drug-resistant myeloid leukemia cells specifically decreased expression of *MDR1*, likely due to a decrease in HuR-bound *MDR1* 3′UTRs, and improved the responsiveness of those cells to adriamycin [49].

In its role as a molecular sponge, *FENDRR* can specifically interact also with proteins. It was shown that *FENDRR* sequesters HuR and this interaction has a stabilizing effect on *FENDRR*, but contributes to the decrease in *MDR1* mRNA levels and restoration of drug sensitivity [49,57]. Of note, *miR-184* competes with HuR for binding to *FENDRR* and marks *FENDRR* for degradation by RISC/Ago [49]. Another protein found to bind to *FENDRR* is Iron-responsive Element Binding Protein 1 (IRP1), discussed later in the context of lung fibrosis [35]. IRP1 binds to *FENDRR-201* and *205* last exon in the position that corresponds to the intron 3/exon 4 junction of *FENDRR-206* or an intron in other isoforms.

### 4.2. Scaffold and Guide

LncRNAs function often as a platform on which certain RNA-binding proteins can be locally increased in abundance and multicomponent complexes assembled to target those ligands to specific genomic locations. *FENDRR* was found physically associated with the chromatin modifying histone methyltransferase complexes such as Polycomb Repressive Complex (PRC) 2 [5,6], TrxG/MLL complex [5,58], and with REST Corepressor (CoREST) 1 protein [6].

PRC2 primarily trimethylates H3 at Lys27 (H3K27me3), causing transcriptional silencing through chromatin compaction. Association of *FENDRR* with PRC2 was identified using RIP-ChIP assay in human fibroblasts (HLFs and human foot fibroblasts (HFFs)) with antibodies against two components of PRC2 complex, SUZ12 or EZH2 [6]. *FENDRR* was also found bound to EZH2 in lung cancer cells CALU1 [59]. Association of *Fendrr* with PRC2 was confirmed using a similar assay in extracts from embryonic mouse cells [5]. In addition, functional analyses in HLFs and HFFs demonstrated that siRNA-mediated depletion of *FENDRR* resulted in de-repression of the PRC2-regulated genes [6]. These results suggest that *FENDRR* not only can bind to chromatin-modifying complexes, but also can guide them to specific genomic locations such as promoters or enhancers. This hypothesis was verified in an in vitro mouse system by Grote et al. [5]. Using pool-down assay, they showed that *Fendrr* directs histone methyltransferase complexes to the *Foxf1* and *Pitx2* promoters, which independently showed a decrease in PRC2 occupancy with loss of *Fendrr*. A 20 nt-long *Fendrr* segment, mapping to mouse *Fendrr* exon 3 was predicted to form a triple-helix with complementary segments of the *Foxf1* and *Pitx2* promoters [5].

REST also represses transcription by forming a complex with CoREST1 and recruiting chromatin-modifying proteins, among others, to methylate H3K9 and demethylate H3K4me or H3K4me2, resulting in chromatin condensation [6]. CoREST proteins are best known for their role as part of the REST complex, but they can also repress target genes in the REST-independent manner [60]. Binding of CoREST1 to *FENDRR* was demonstrated in HLFs and HFFs using RIP-ChIP assay and antibodies against CoREST1 [6].

In contrast to the repressive PRC2 and CoREST complexes, TrxG/MLL proteins are responsible for transcription-activating methylation of H3, H3K4me3. The physical interaction of TrxG/Mll complex with *Fendrr* was identified using a pool-down assay in the murine embryonic cell extracts with antibodies against a component of this transmethylase complex, WDR5 [5].

## 5. *FENDRR* in Development and Disease

### 5.1. FENDRR Expression during Development

Grote et al. [5] reported that *Fendrr* expression in mice is confined to the nascent lateral plate mesoderm. They found expression of *Fendrr* in cardiac mesoderm progenitor cells at the early midstreak stage E6.5–7. Sauvageau et al. [61] found that in mice developing respiratory and digestive tracts at E14.5, *Fendrr* is expressed in the pulmonary mesenchyme surrounding the bronchiolar epithelial cells, in mesenchymal cells of the developing mucosa, in the muscular layer of the gut, and in the lymphoid aggregations within mucosa of the gut. Lai et al. [62] showed that in E12.5 embryos, *Fendrr* is expressed in the frontonasal process, upper respiratory track, lungs, and the posterior aorta-gonad-mesonephron region.

Described by Grote et al. [5], homozygous loss of *Fendrr* was lethal at E13.75 due to heart and omphalocele defects. Sauvageau et al. [61] also showed that homozygous loss of *Fendrr* is lethal although at a later, perinatal stage; *Fendrr*^−/−^ lungs at the pseudoglandular stage (E14.5) were hypoplastic, had decreased number of pulmonary arteries, and featured general failure of vasculogenesis. At E18.5, *Fendrr*^−/−^ lungs had fewer and enlarged alveoli. The observed respiratory failure at birth was likely caused by the defects in lung maturation and vascularization. Regarding development of the esophagus and the gut, E18.5 embryos featured thinning of the mesenchymal layer of the mucosa and external smooth muscle layers in the esophagus. Moreover, they featured intraventricular septal heart defects prior to birth. Perinatal lethality in *Fendrr*^−/−^ mice was also reported by Lai et al. [62]. E13.5 embryos had smaller lungs with globular and disorganized lobes. Mice died shortly after birth due to breathing problems. Some phenotypic differences between *Fendrr* knockout mice described by the three groups may be due to differences in the *Fendrr* ablation strategies used. While Grote et al. [5] eliminated *Fendrr* transcription by replacing the 1st exon of *Fendrr* with multiple transcription terminators, the other two groups [61,62] replaced most of the *Fendrr* gene with the *lacZ* reporter while retaining the transcription from the endogenous *Fendrr* promoter.

In the adult mouse, *Fendrr* is highly expressed in the lungs and colon, then in the liver, spleen, brain, and testes (http://useast.ensembl.org/Mus_musculus) [61]. In humans, gene expression pattern determined using RNA-seq in 948 normal adults (the NIH Genotype-Tissue Expression (GTEx) project) showed the highest *FENDRR* levels in the bladder, lungs, esophagus, colon, prostate, and small intestine [63]. Similar results were independently obtained by other group using RNA-seq of transcriptomes from 27 different tissue samples from 95 normal adults [64]. These studies showed that *FENDRR* has the highest expression in the lungs, followed by urinary bladder, gall bladder, esophagus, prostate and appendix, and was almost undetectable in the heart, kidney, and liver.

### 5.2. Alveolar Capillary Dysplasia with Misalignment of Pulmonary Veins (ACDMPV)

The involvement of *Fendrr* in mouse lung development and the regulation of *FENDRR* expression by FOXF1 as well as by the *FOXF1* enhancer suggest that *FENDRR* might be involved in etiology of ACDMPV and/or associated diseases. Point mutations involving *FOXF1* and CNV deletions of *FOXF1* or its enhancer are causative for ACDMPV in more than 80% of patients.

To determine whether any genes or pathways affected in ACDMPV lungs could be controlled by *FOXF1* through *FENDRR*, we have performed RNA-seq in *FENDRR*-depleted (by siRNA or CRISPR/Cas9) IMR-90 cells [65]. Our preliminary ConsensusPathDB-based analyses of genes whose expression changed following *FENDRR* depletion in IMR-90 cells suggest deregulation of some pathways also affected in the ACDMPV lungs, including those involved in RNA processing, cell cycle regulation, membrane trafficking, VEGFA-VEGFR2 signaling, TGF-β signaling, EGF signaling, β-catenin-independent WNT signaling, G protein signaling, and other processes [65]. Genes involved in vascular development, similarly affected in ACDMPV and *FENDRR*-depleted fibroblasts, include, e.g., *PDGFRA*, *PLXNC1*, or serotonin transporter genes *SLC5A1* and *SLC6A4* with roles in vascular remodeling [65]. Genes that were found upregulated in ACDMPV and *FENDRR*-deficient fibroblasts include, e.g., *COL6A2* and *ELN* which might contribute to pulmonary arterial hypertension [65].

### 5.3. Cancer

Long non-coding RNAs have been shown to be involved in the pathogenesis of various malignant cancers as tumor suppressors or oncogenes [66,67,68]. *FENDRR* was proposed to have a tumor suppressor role by inhibiting cell proliferation, motility, chemotherapy resistance, or inducing apoptosis. Its expression level was found often significantly reduced in several cancers, including lung adenocarcinoma and squamous cell carcinoma, bladder urothelial carcinoma, or colorectal adenocarcinoma [33,39,40,41,42,46,47,48,50,51,52,53,54,55,56,69]. On the other hand, *FENDRR* overexpression correlated with a decreased rate of proliferation of cancer cell lines [54,70], inhibition of invasive capacity of Non-small Cell Lung Cancer (NSCLC) cells and a sharp decline in tumor growth in vitro [54]. The A549 and PC-9 lung cancer cells with induced high expression of *FENDRR* exhibited significantly decreased growth rate of tumor xenograft compared with their controls in vitro. Xu et al. [71] have reported that *FENDER* over-expression also suppressed migration and invasion of gastric cancer cells.

*FENDRR* has been proposed to play an inhibitory role in cancer by sponging miRs that target mRNAs of tumor suppressors (Table 1). *FENDRR* was shown to sequester miR-761 leading to an increase in metalloproteinase inhibitor TIMP2 level and thus inhibition of cell invasion [54,55]. In gastric cancer, *FENDRR* decrease increased cell migration and invasion in vitro through upregulation of metalloproteinase genes *MMP2* and *MMP9* involved in degradation of extracellular matrix [71]. *FENDRR* also targets oncogene promoters with, e.g., chromatin modifying PRC2 complex, leading to a decrease in the expression of those oncogenes. Loss of *FENDRR*, e.g., induced cell proliferation, epithelial–mesenchymal transition, and metastasis of NSCLC cells via EZH2 and downregulation of *FOXF1* [59].

Lastly, *FENDRR* was linked to regulation of cancer cell drug resistance. It was found to be dramatically decreased, e.g., in the doxorubicin-resistant osteosarcoma cell lines and tissues [72]. *FENDRR* apparently acts as a suppressor of chemoresistance by downregulating multidrug resistance genes, coding for ABC-transporters ABCB1, ABCC1 [72], and ABCC10 [70]. The overexpression of *FENDRR* correlated with suppression of doxorubicin resistance of osteosarcoma cells, cell cycle progression, and promoted cell apoptosis [72].

### 5.4. Fibrosis

Idiopathic Pulmonary Fibrosis (IPF) is caused by uncontrolled stimulation of fibroblasts, leading to the accumulation of ACTA2, collagen and fibronectin in the extracellular matrix, resulting in loss of tissue architecture and functioning [73,74]. The disease is incurable with a life expectancy of 3–5 years. *FENDRR* level was found to be decreased both in mesenchyme of fibrotic human lungs, likely due to the increased TGF-β-induced SMAD3 signaling [35,75], and in sera of lung fibrosis patients [76]. *FENDRR* shows anti-fibrotic effect in lung fibroblasts by (i) sponging fibroblast activating *miR-214* (it contains six computationally predicted binding sites for this miR), and (ii) reducing iron level through interaction with IRP1 [35]. Iron is thought to activate pro-fibrotic TGF-β signaling to SMAD3 which inhibits *FENDRR* expression and activates expression of collagen and other extracellular matrix protein genes. IRP1 binds, among others, to iron-responsive element of Transferrin Receptor *TFRC* mRNA (TFRC is needed for the import of iron ions in the ferric form into cells [77]), allowing for its translation. *FENDRR* and *TFRC* mRNA compete for IRP1 so that in the presence of *FENDRR*, more *TFRC* mRNA remains unbound to IRP1 and is rapidly degraded leading to the depletion of intracellular iron level.

However, the anti-fibrotic functioning of *FENDRR* can be tissue specific. In a mouse fibrotic heart, *Fendrr* level rather increased [44]. *Fendrr* seems to play pro-fibrotic role in the developed heart by sponging *miR-106b*, which targets pro-fibrotic *Smad3* mRNA for degradation.

### 5.5. Inflammation

*Macrophage polarization*: *FENDRR* was identified as a positive regulator of pro-inflammatory M1 macrophage polarization via the STAT1-dependent pathway [78]. Disorders associated with macrophage polarization include fibrosis [79], cancer [80], infections [81], insulin resistance [82], atherosclerosis [83], and autoimmune disease [84]. The exact mechanism of M1 polarization is unknown. *FENDRR* could function in this process as a chromatin modifier, increasing the expression of pro-inflammatory *CXCL10* through TrxG/MLL-mediated H3K4me3 methylation, or EZH2-dependent H3K27 methylation that suppresses anti-inflammatory genes. It is also possible that *FENDRR* reduces iron level in macrophages, leading to activation of STAT1 signaling and M1 macrophage polarization.

*Pancreatitis*: In an in vitro rat model of caerulein-induced inflammation of the pancreas, pancreatitis, ANXA2 and *FENDRR* were found to be increased in pancreatic acinar cells [32]. *FENDRR* and Anxa2 upregulation correlated with an increase in pro-apoptotic BAX and a decrease in an anti-apoptotic BCL2. *FENDRR* was also found bound to ANXA2 and this interaction might contribute to apoptosis.

### 5.6. Cardiovascular Disorders

*FENDRR* was shown to be essential for heart development in mice [5,61,62]. However, *FENDRR*’s role in cardiovascular development in humans is unknown. *FENDRR* was suggested to have cardio-protective role based on negative correlation of its levels in peripheral blood mononuclear cells with left ventricular mass index in hypertensive patients [85].

## 6. Concluding Remarks

The lncRNA *FENDRR* emerges as a significant cell type- and developmental stage-specific regulator of gene expression at transcriptional and post-transcriptional levels. Its own expression involves promoter activation both by a distant tissue-specific enhancer acting *in cis*, shared with *FENDRR*-neighboring *FOXF1*, and by *trans*-acting FOXF1. This kind of expression control may represent a paradigm for the regulation of other divergent gene pairs. However, the overall current understanding of *FENDRR* structure and functioning is just the tip of the iceberg. For instance, *FENDRR* spatial structure is unknown and likely depends on its multiple ligands and local environment. This picture is further complicated by the existence of numerous *FENDRR* isoforms, whose presence may be tissue- and developmental stage-dependent. In addition, *FENDRR* subcellular localization requires further clarification. In mouse embryonic lungs, it seems mostly nuclear, whereas in adult human lung fibroblasts, it localizes preferentially to the cytoplasm. Moreover, *FENDRR*’s known ligands have been identified in in vitro experiments focused rather on testing selected proteins or miRs. However, the current knowledge already points at the importance of *FENDRR* in development and tissue maintenance. In vitro experiments have shown that increasing *FENDRR* expression can potentially be of therapeutic significance in many cancers and lung fibrosis. Nevertheless, although targeting cancer cells and fibroblasts with *FENDRR* can be beneficial, *FENDRR* overexpression in epithelia may inhibit epithelial cell proliferation and hinder restoration of organ function. Thus, therapeutic manipulation with *FENDRR* expression would have to be organ/tissue specific. Other challenges faced in *FENDRR* studies are related to its very nature as a rapidly evolving regulatory molecule or even group of molecules, taking into account all its alternatively spliced isoforms that sometimes may be involved in contradictive biological processes.

## Figures and Tables

**Table 1:** MicroRNA–*FENDRR* interactions affected in cancer, fibrosis and other diseases.

MicroRNA Binding to *FENDRR*		MicroRNA Target		Disease Association		Reference
MicroRNA	MicroRNA Binding Site on *FENDRR* (hg38)	Gene	Function	Disorder	*FENDRR* Expression	
*miR-15a-5p*	Exon 4: 86,488,730–86,488,724	*TUBA1A*	Suppresses cell proliferation and viability	Cervical cancer	Decreased	[39]
*miR-15b-5p*	Exon 4: 86,488,743–86,488,723					
*miR-18a-5p*	Exon 4: 86,488,853–86,488,840	*IRF2*	Pro-apoptotic, suppressor of cell migration and proliferation	Non-small cell lung cancer	n/a	[40]
		*ING4*	Growth inhibitor	Colorectal cancer	Decreased	[41]
		*RUNX1*	Pro-apoptotic TF, suppressor of cell proliferation, invasion and migration	Prostate cancer	Decreased	[42]
		*NOTCH2*	Pro-Endothelial–Mesenchymal Transition (EndMT), contributes to cardiac fibrosis	Hyperglycemia-induced EndMT and cardiac fibrosis	n/a	[43]
*miR-106b* (mouse)	Mouse *Fendrr* exon 5	*Smad3*	Inhibits *FOXF1* expression, pro-fibrotic	TAC-induced cardiac fibrosis	Increased	[44]
*miR-126*	Exon 1: 86,509,005–86,508,980	*VEGFA*	Angiogenesis, apoptosis	Hypertensive intracerebral hemorrhage	Increased	[45]
			Positively regulates multidrug resistance gene *MRP1*	Non-small cell lung cancer	n/a	[46]
		*EGFL7*	Tumor angiogenesis	Hepatocellular carcinoma	n/a	[47]
		*SOX2*	Positively regulates expression of *PLAC1*	Gastric cancer	n/a	[48]
*miR-184*	Exon 4: 86,487,935–86,487,917	*MDR1* (*ABCB1*)	Drug transporter (*miR-184* competes with HuR for *FENDRR*; *FENDRR* + HuR − MDR1; HuR > MDR1	Chronic myelogenous leukemia; chemotherapy resistance	Decreased	[49]
*miR-214-3p*	Exon 4: 86,488,737–86,488,725	*TET2*	DNA demethylation (loss of *TET2* leads to hypermethylation of tumor suppressor *RASSF1A* promoter	Gastric cancer	Decreased	[50]
		*PTEN*	Tumor suppressor (negative regulator of Akt/PKB signaling)		n/a	[51]
	Six binding sites in exon 4	n/a	n/a	Lung fibrosis	Decreased	[35]
*miR-362-5p*	Exon 4: 86,487,984–86,487,963	*NPR3*	Suppresses p38-MAPK signaling	Hepatocellular carcinoma cells	Decreased	[52]
*miR-423-5p*	Exon 3: 86,490,399–86,490,388	*GADD45B*	Inhibits cell proliferation and induces apoptosis	Hepatocellular carcinoma	Decreased	[53]
*miR-424*	n/a	*FOXF1*	Suppressor of cell proliferation	Lung cancer	Decreased	[33]
*miR-761*	Exon 4: 86,488,737–86,488725	n/a	Suppressor of cell motility and invasion	Non-small cell lung cancer	Decreased	[54]
		*TIMP2*	Inhibitor of matrix metalloproteinases (MMPs) and cell invasion	Non-small cell lung cancer	Decreased	[55]
*miR-148a* *miR-195* *miR-196b* *miR-301b*	n/a	*EPAS1*	*FENDRR*-miR-mRNA potential associations deduced based on comparative gene expression analysis of TCGA-lung adenocarcinoma datasets	Non-small cell lung cancer	Decreased	[56]
		*EDNRB*				
		n/a				
		*SOX21*				
